# The Response of Spore Germination of *Sphagnum* Mosses to Single and Combined Fire-Related Cues

**DOI:** 10.3390/plants11040485

**Published:** 2022-02-11

**Authors:** Shuayib Yusup, Sebastian Sundberg, Beibei Fan, Mamtimin Sulayman, Zhao-Jun Bu

**Affiliations:** 1 Key Laboratory of Geographical Processes and Ecological Security in Changbai Mountains, Ministry of Education, School of Geographical Sciences, Northeast Normal University, Renmin 5268, Changchun 130024, China; xiayp680@nenu.edu.cn (S.Y.); fanbb240@nenu.edu.cn (B.F.); 2 State Environmental Protection Key Laboratory of Wetland Ecology and Vegetation Restoration, Institute for Peat and Mire Research, Northeast Normal University, Renmin 5268, Changchun 130024, China; sebastian.sundberg@slu.se; 3 Jilin Provincial Key Laboratory for Wetland Ecological Processes and Environmental Change in the Changbai Mountains, Renmin 5268, Changchun 130024, China; 4 Xinjiang Key Laboratory of Biological Resources and Genetic Engineering, College of Life Science and Technology, Xinjiang University, Urumqi 830046, China; 5 Evolutionary Biology Centre, Department of Plant Ecology and Evolution, Uppsala University, Norbyvägen 18D, SE-752 36 Uppsala, Sweden; 6 SLU Swedish Species Information Centre, Swedish University of Agricultural Sciences, SE-750 07 Uppsala, Sweden

**Keywords:** spore germinability, fire disturbance, persistence, germination cue

## Abstract

Plants in flammable ecosystems have different response strategies to fire, such as increasing germination after exposure to smoke and break of dormancy through heat shock. Peatlands are ecosystems that are more likely to be disturbed by fire with increasing temperatures, but it is not clear how fire affects spore germination of *Sphagnum*, the dominant plants in peatlands. Here, we hypothesize that *Sphagnum* spores respond positively to single and combined treatments of moderate heat and smoke (by increased germinability), while spore germinability decreases in response to high temperature. We exposed the *Sphagnum* spores of four selected species (*S. angustifolium*, *S. fuscum*, *S. magellanicum* and *S. squarrosum*) collected from peatlands in the Changbai Mountains to heat (40, 60 and 100 °C), on its own and combined with smoke-water treatments. Our results showed that a heat of 100 °C inhibited the spore germination or even killed spores of all species, while spore germination of three (*Sphagnum* *angustifolium*, *S. fuscum* and *S. squarrosum*) of the four species was promoted by 40 and 60 °C heat compared to the control (20 °C). Hollow species (*S. angustifolium* and *S. squarrosum*) showed a stronger positive responsive to heat than hummock species (*S. fuscum* and *S. magellanicum*). *Sphagnum* *fuscum* spores responded positively to the combined heat and smoke treatment while the other species did not. For the first time, we demonstrate the positive effects of heat on its own and in combination with smoke on spore germination in wetland mosses, which may be important for the establishment and persistence of peatmoss populations after fire.

## 1. Introduction

Many ecosystems depend on fire to maintain stability and their adaption to fire may determine their fate under the background of climate warming [1,2,3]. The survival and reproduction of plants can be directly affected by fire. Therefore, in the long-term evolution process, vascular plants in fire-prone ecosystems have formed a series of morphological and physiological fire-adapted traits. For example, pine species in North America can tolerate frequent lightning fires by forming a thick bark and delaying cone cracking [4]; The seeds of Australian *Xanthorrhoea gracilis* Endl. buried in soil can tolerate wildfires with a surface temperature of up to 300 °C and immediately germinate and flower [5]; germination of the epiphytic orchid, *Oberonia ensiformis* (J. E. Smith) Lindl, is strongly promoted by smoke, and its germination percentage is otherwise zero in the absence of smoke [6]. The fire adaptation strategy of plants makes the populations survive and develop continuously, and the fire-related renewal strategies, such as the response characteristics of propagules (vegetative or sexual) to fire factors, are particularly important for vegetation recovery and change of community structure after fire [7].

Fire-stimulated germination is found in many seed plants from fire-prone ecosystems [8,9]. Post-fire germination may be triggered by different mechanisms, with heat and smoke being the main fire-related germination cues [10]. Heat can improve germination by accelerating after-ripening in species with a water-permeable seed coat, or by rupturing the seed coat structure, allowing water uptake, in species with water impermeable seeds [11,12]. The smoke produced during fire can also stimulate germination and seedling growth [9,13,14,15]. The mechanism is that smoke contains active compounds (nitrogen oxides, glyceronitrile and butenolide), similar to the plant hormones gibberellin or cytokinin, which can promote embryo development and break seed dormancy [9,16,17,18].

In natural communities, smoke and heat often act together, but the way of action varies according to the location of seed distribution [19]. In general, heat mainly impacts the surface layer of the soil and the seeds remaining in the lower parts of the branches [19], the aerosol smoke mainly affects seeds remaining in the higher parts of the branches [20], while seeds deep in the soil are affected by the interaction of heat and aqueous smoke [9]. In addition, many studies show that smoke cannot stimulate germination of seeds that are physically dormant; therefore, smoke promotion in species with water-impermeable seeds is likely to occur only if seeds are released from physical dormancy by suitable environmental factors [12,21]. Thus, the response of the plant propagules (seeds and spores) may be very complex and is dependent on several factors including the interaction between fire-related cues such as heat and smoke, the depth at which seeds are buried, and a series of morphological and physiological traits of propagules [19,22].

Up to now, research on the impact of fire on vegetation succession and regeneration has mostly been concentrated on ecosystems around the Mediterranean region [8], Africa [23], Australia [24] and California in North America [1], but the effect of wildfires on non-fire prone ecosystems, such as peatlands, is poorly known [25]. Generally, peatlands are not prone to natural fires due to waterlogging. However, studies on charcoal from Canada [26] and Poland [27] have also shown that fire is an important disturbance in northern peatlands.

Peat mosses, namely the genus of *Sphagnum* are the dominant and most important carbon sequestering plants in northern peatlands [28]. Many *Sphagnum* species regularly produce spores every year, and most spores will be buried in the stratum, with the accumulation of peat, to form a spore bank [29,30,31]. Since the surface vegetation and/or soil has good thermal buffering during wildfires, spores in deep peat layers can avoid direct damage [29,32]. Mechanisms of bryophyte regeneration and colonization by gametophytes following natural disturbances, particularly fire, have been documented in bogs [33,34,35] and in rich fens [36]. However, it is not clear whether peatland bryophyte (especially *Sphagnum*) spores will respond to fire and its related cues.

Recently, the role of fire for potential regeneration of peatland bryophytes from spores has been addressed in some studies [32,37], and especially the promotive effect of smoke on spore germination has been demonstrated [37]. We, however, still lack a comprehensive understanding in how heat alone or its combination with smoke affect the germination of bryophyte spores. In this study, our aim was to test the following hypotheses: (1) Heat will improve the germination of *Sphagnum* spores; (2) the positive effects of heat on spore germination may be more pronounced when interacting with smoke; and (3) since spores of hollow species (*S. angustifolium* and *S. squarrosum*) are more susceptible to fire than those of hummock species (*S. fuscum* and *S. magellanicum*) during dry periods, the spores of hollow species may show a more accentuated response than hummock species to heat and smoke.

## 2. Results

### 2.1. Effects of Heat on Spore Germination

#### 2.1.1. Germination Percentage (GP)

**Figure 1:**
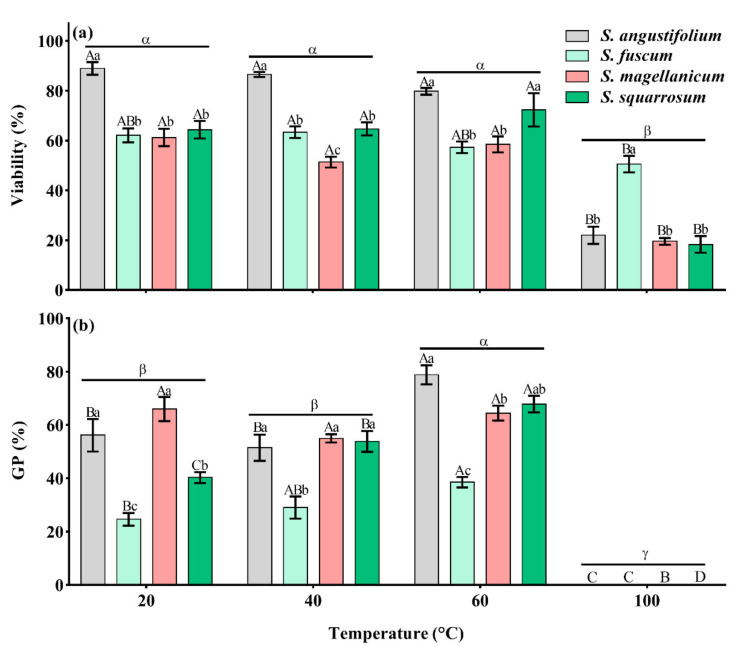
Effects of different heat temperatures on spore (**a**) viability (dyeing percentage) (**b**) germination percentage (GP) and in four *Sphagnum* species. Error bars represent SEM (*n* = 3). Lowercase letters (a, b and c) represent significant difference among species at the same heat temperature and uppercase letters (A, B, C and D) represent significant difference among heat temperatures in the same species (also in others). “α”, “β” and “γ” represent significant difference among the total germination percentage (21d) and viability after heat treatment.

**Figure 2:**
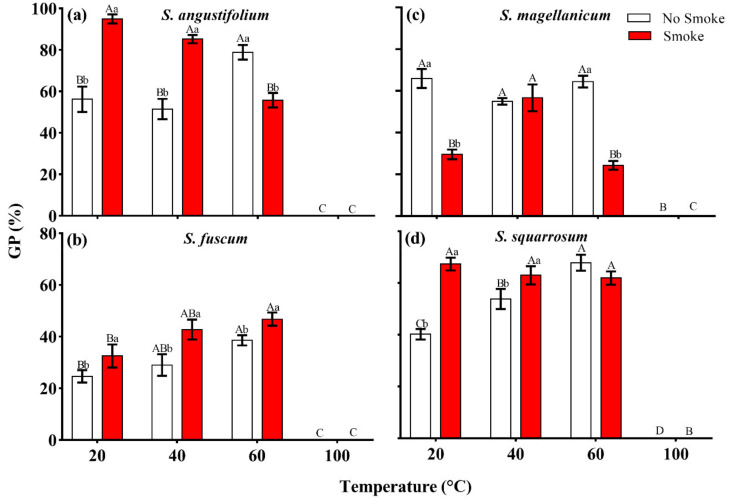
Interactive effect between smoke–water and heat on spore germination percentage (GP) in four *Sphagnum species* ((**a**), *Sphagnum angustifolium*; (**b**), *S. fuscum*; (**c**), *S. magellanicum*; (**d**), *S. squarrosum*). Error bars represent SEM (*n* = 3). Lowercase letters (a, b and c) represent significant difference among smoke–water treatments at the same heat-shock temperature and uppercase letters (A, B, C and D) represent significant difference among heat temperatures at the same smoke–water treatment.

There were significant differences in spore germination among the four *Sphagnum* species (*p* < 0.001, Table 1). The GP of *S. fuscum* and *S. squarrosum* was lower, 24.6 ± 2.4% and 40.3 ± 2.0% (Figure 1b and Figure 2b,d), while the GP of *S. angustifolium* and *S. magellanicum* was relatively high, 56.2 ± 6.1% and 66.0 ± 4.5%, respectively (Figure 1b and Figure 2a,c). Heat had a significant effect on spore germination, showing a unimodal response to increasing temperature (*p* < 0.001) (Table 1; Figure 1b). There were interspecific differences in the germination behavior (*p* < 0.001) (Table 1; Figure 1b). The spore GPs of *S. angustifolium*, *S. fuscum* and *S. squarrosum* increased with moderate heat temperature (40 °C and 60 °C) (*p =* 0.017 for *S. angustifolium*; *p* = 0.031 for *S. fuscum*; *p* = 0.002 for *S. squarrosum*) and reached a maximum when treated with 60 °C (10 min), 38.5 ± 2.0%, 78.8 ± 3.5% and 67.9 ± 3.1%, respectively (Figure 1b and Figure 2a,b,d). However, there was no difference in spore germination of *S. magellanicum* between heat of 40 °C and 60 °C and the control (*p* = 0.10, Table 2; Figure 1b and Figure 2c). Heat at 100 °C significantly inhibited spore germination of all species (*p* < 0.001) (Figure 1b and Figure 2). 

#### 2.1.2. Germination Speed (GS) and Heat Treatment Effect Index (TEI)

**Figure 3:**
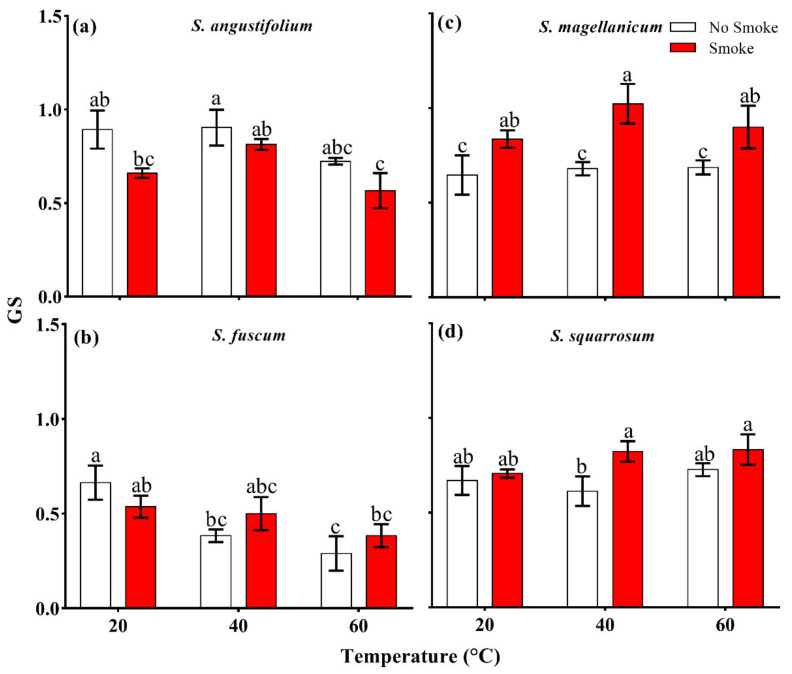
Interactive effect between smoke–water and heat on spore germination speed (GS) in four *Sphagnum* species. ((**a**), *Sphagnum angustifolium*; (**b**), *S. fuscum*; (**c**), *S. magellanicum*; (**d**), *S. squarrosum*). Error bars represent SEM (*n* = 3). Different lowercase letters represent significant differences (*p* < 0.05) between the treatments (smoke–water and heat treatments singly and combined).

After heat treatments (40 °C and 60 °C), there were no differences in GS in *S. angustifolium*, *S. magellanicum* and *S. squarrosum* compared with the controls (*p* > 0.05, Table 2; Figure 3a,c,d), while the GS of *S. fuscum* clearly decreased (*p* = 0.031, Table 2; Figure 3b).

**Figure 4:**
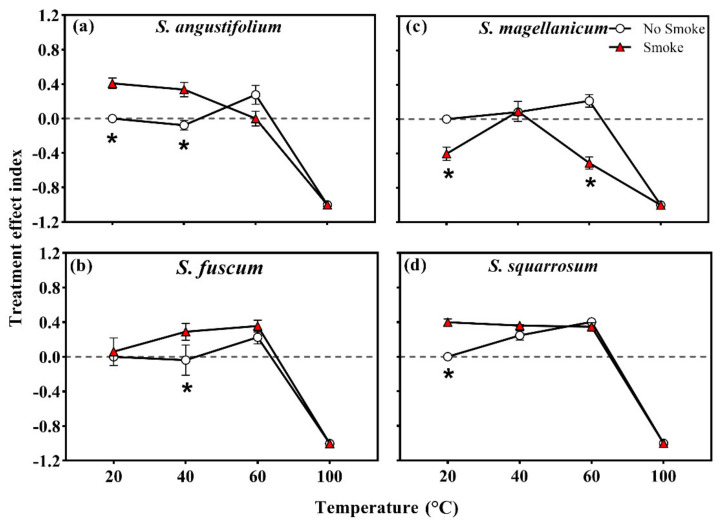
Treatment effect index (TEI) of four *Sphagnum* species ((**a**), *Sphagnum angustifolium*; (**b**), *S. fuscum*; (**c**), *S. magellanicum*; (**d**), *S. squarrosum*) after single and combined treatment of heat temperature and smoke–water. Error bars represent SEM (*n* = 3). Different lowercase letters represent significant differences (*p* < 0.05) between heat treatments. Asterisks represent significant differences (*p* < 0.05) among smoke–water treatments at the same heat-shock temperature.

The heat treatment effect index (TEI) of *S. angustifolium* at 60 °C was higher than that of the control group (*p* = 0.028; Table 2; Figure 4a), while that at 40 °C did not differ. The TEI of *S. squarrosum* was 0.25 ± 0.05 (40 °C) and 0.40 ± 0.03 (60 °C), both greater than that of the control group (*p* = 0.049 for 40 °C; *p* < 0.001 for 60 °C) (Table 2; Figure 4d). Compared to the control group, the TEI of *S. fuscum* and *S. magellanicum* did not change after heat treatment at 40 °C and 60 °C (*p* = 0.260 for *S. fuscum*; *p* = 0.056 for *S. magellanicum*) (Table 2; Figure 4b,c).

#### 2.1.3. Spore Viability

From the methylene blue dyeing of spores, there was a significant difference in the initial spore viability among the four *Sphagnum* species (*p* < 0.001) (Table 1; Figure 1a). The initial viability of *S. angustifolium* was the highest (88.9 ± 2.6%), and the viability of *S. magellanicum* was the lowest (61.3 ± 3.5%) (Figure 1a). After heat treatment, the viability of all tested species decreased significantly (*p* < 0.001 for *S. angustifolium*, *S. magellanicum* and *S. squarrosum*; *p* = 0.027 for *S. fuscum*) (Table 1; Figure 1a) mainly because of the strong negative effect of 100 °C; There was no significant difference in spore viability between treatments at 40 °C, 60 °C and the control group (Figure 1a).

### 2.2. Effects of Heat + Smoke on Spore Germination

#### 2.2.1. Germination Percentage

The germination was significantly affected by the combined treatment of heat and smoke-water in all four tested species (*p* < 0.01, Table 2; Figure 2). Compared to the control group (20 °C + no smoke), the GP of *S. angustifolium* increased significantly under the combined treatment of 40 °C + smoke (*p* < 0.001, Table 2; Figure 2a). Except 100 °C + smoke treatment, spore germination of *S. fuscum* increased in all combined treatments (*p* = 0.007) and reached the highest value (46.7 ± 2.6%) at 60 °C + smoke (Table 2; Figure 2b). In *S. magellanicum*, GP was highest in the control group (66.0 ± 4.5%) and, besides from 40 °C + smoke, all other smoke treatments decreased its germination (*p* < 0.001) (Table 2; Figure 2c). Compared to the control group, both 40 °C + smoke and 60 °C + smoke clearly promoted spore germination in *S. squarrosum* (*p* < 0.001, Table 2; Figure 2d).

#### 2.2.2. Germination Speed and Heat Treatment Effect Index

The GS in *S. angustifolium* and *S. fuscum* decelerated under the combined treatment of 60 °C + smoke (*p* < 0.05 for both, Figure 3a,b). In *S. magellanicum*, the combined treatments of 40 °C + smoke and 60 °C + smoke obviously accelerated the GS (*p* < 0.05, Figure 3c). 

In terms of the treatment effect index (TEI), *S. angustifolium*, *S. fuscum* and *S. squarrosum* showed positive values in the combined treatments of 40 °C + smoke and/or 60 °C + smoke (*p* < 0.05 for all, Table 2; Figure 4a,b,d). In *S. magellanicum*, the TEI was negative in the combined treatment of 60 °C + smoke (*p* = 0.001, Table 2; Figure 4c).

## 3. Discussion

### 3.1. Heat and Spore Germination

The results show that *Sphagnum* spores can tolerate temperatures of up to 60 °C, and that temperatures of 40 °C or 60 °C actually stimulate spore germination, which verifies our first hypothesis. Generally, seeds with a thick and water-impermeable coat tend to respond positively to heat shock [12,38]. The mechanism is that high temperatures stimulate germination by rupturing the seed coat, allowing water uptake, in species with otherwise water-impermeable seeds. The dormancy-breaking temperatures of these seeds are generally relatively high [11], with temperature ranges of 80–100 °C [39]. Williams et al. [24] found that a short-term high temperature treatment at 80–100 °C can break the seed dormancy of several plant species, thereby stimulating seed germination. Zhang et al. [40] found that *Dodonaea viscosa* seeds have an obvious heat response at temperature above 40 °C, and that germination reaches its maximum after treatment at 80 °C (for 10 min). In contrast, the temperature threshold (40 °C and 60 °C) that can stimulate *Sphagnum* spore germination in this study is relatively low. This indicates that high temperatures do not play a mechanical role in rupturing the spore wall, like in seeds, but is more likely to break dormancy by affecting the physiological process of spores [41].

### 3.2. Effect of Smoke on Spore Germination

Keely et al. [42] and Baldwin et al. [43] provided evidence that trace gases in smoke such as nitrogen dioxide (NO_2_) and potentially nitric oxide (NO) are responsible for induced germination and dormancy release of annuals in chaparral. Although NO is recognized as a plant signaling compound [44] and a promoter of seed germination [43], the proposed role of NO*x* as the dormancy-breaking cue in plant-derived smoke (PDS) has been challenged by several studies. Preston et al. [45] found that smoke-responsive plant species did not respond to NO*x* generated from solutions of sodium nitroprusside, and the stimulatory effect of smoke could not be inhibited with a specific nitrogen-oxide scavenger [46]. Flematti et al. [16], in 2004, isolated the predominant chemical compound in smoke-water and identified as karrikinolide (KAR_1_: 3-methyl-2*H*-furo[2,3-*c*]pyran-2-one), and it was considered as the most recognized active component that promotes seed germination [9]. Although there is no unified understanding of the substances in smoke–water, it is still believed that the KAR_1_ and NO*x* in PDS and its solution may promote the germination of many plant seeds at the same time or different seeds respectively. In this study, although we did not report whether the single effect of smoke–water can promote spore germination, from the more significant results of heat treatment (20 °C, 40 °C and 60 °C) + smoke on spore germination of three *Sphagnum* species (*S. angustifolium*, *S. fuscum* and *S. squarrosum*) compared with the control group and corresponding heat treatment, we can get that smoke–water does have a certain promotive effect on spore germination (Figure 2). In addition, our previous study also clearly proved that smoke–water can strongly promote the germination of ten bryophyte spores [37]. In a certain sense, smoke and/or heat + smoke responsiveness of spores could not only be useful to remind that basic features of dormancy-breaking treatments, but also fulfill the knowledge gap in the bryophyte dormancy and reveal the not yet known mechanisms to initiate dormancy-breaking of spores [47].

### 3.3. Spore Germination in Relation to Heat and Smoke–Water

Under natural conditions, smoke and heat production during combustion are basically synchronous. Therefore, the effect of fire on propagule germination is considered to be the result of the interaction between heat and smoke [10]. In this experiment, germination of *S. fuscum* after heat treatment (40 °C and 60 °C) + smoke–water was higher than after the single treatment of heat (Table 2; Figure 2). This is consistent with our second hypothesis and do agree with previous findings in seed germination of some species. For example, Tieu et al. [22] studied the germination of seven naturally distributed plant seeds in South Australia under the combined treatment of heat and smoke and found that germination of *Sowerbaea laxiflora* seeds did not change when exposed to heat, while it increased when treated with heat + smoke. Zirondi et al. [38] found that after exposure to 100 °C + smoke-water, the germination of almost all tested legume species with impermeable seed coats increased significantly compared to the control, and single treatments by smoke and heat. However, combined treatments were not more effective than single heat or smoke-water treatment for stimulating germination of spores in the three other species (*S. angustifolium*, *S. magellanicum* and *S. squarrosum*). This is similar to the results of studies by Abella et al. [48] and Figueroa et al. [49] who found that there was no more effective stimulation of seed germination after the combined treatments of smoke and heat than after single smoke or heat treatments.

### 3.4. Fire-Responsive Germination and Species Habitat Preference

Plant propagule (seed and spore) dormancy and germination characteristics may vary depending on habitats and species’ life history strategies [50,51,52]. A previous study showed that species indicating disturbance and those preferring soils more or less rich in nitrogen respond more strongly to fire related cues [51]. Naturally, reflecting microtopographical differences, hollow species are more vulnerable to fire than hummock species in dry periods [53,54]. In this study, the tested hollow species (*S. angustifolium* and *S. squarrosum*) showed a strong positive response to smoke-water. However, the response of hummock species to smoke was inconsistent. *S. fuscum* showed a positive response but not as strong as the hollow species, *S. magellanicum* showed a negative response (Figure 1). Moderate heat treatment (40 °C and/or 60 °C) had an effect on all four tested species, but the stimulation of germination was higher in hollow than in hummock species. In addition, the combined heat (40 and/or 60 °C) + smoke treatment significantly stimulated spore germination in the two hollow species, while the effect on the hummock species was more variable (Figure 2). This result supports our third hypothesis, that the spores of hollow species are more adapted to fire than the hummock species. The difference in response of different species to heat and smoke may be an important mechanism for the co-existence of multiple species in these northerly peatlands.

### 3.5. Fire Disturbance and Establishment of Sphagnum Spores

Bryophytes are generally considered as pioneer species in the process of post fire secondary succession, but their specific re-establishment mechanism is not clear [35]. Some scholars believe that the spore bank is important for the re-establishment of *Sphagnum* populations after catastrophic disturbance [31,55,56]. In this study, we found that low-intensity heat and smoke–water, on its own and in combination, can stimulate germination of *Sphagnum* spores. In Hani peatland of the Changbai Mountains, the temperature can easily reach 40 °C or even higher [32], which is beneficial for the germination and establishment of *Sphagnum* spores. During a fire, the surface temperature of a peatland will exceed 100 °C, but at a depth of only 1–5 cm, the surface moss layer and peat acts as effective insulators to moderate the temperature increase, and buried spores can retain their viability [32]. In addition, our experiment also indicates that *Sphagnum* spores have a certain tolerance to high temperature (40–60 °C). Thus, adaptable heat temperatures in deep peat layers may be one important reason and condition for the activation of the persistent spore bank of *Sphagnum* mosses, for re-establishment after a fire.

Combustion is a complex process. In addition to generating a large amount of heat energy, it will also cause changes in many habitat conditions, such as soil structure, nutrient status, light conditions, water level and microtopography [25,57,58]. Therefore, in the future research, we should comprehensively consider the various effects of fire to reveal the mechanisms of fire affecting bryophyte regeneration in peatlands.

## 4. Materials and Methods

### 4.1. Study Species, Spore Capsule Collection and Spore Suspension Preparation

Two hummock species, *Sphagnum fuscum* (Schimp.) Klinggr. and *S. magellanicum* Brid., and two hollow species, *S. angustifolium* C. Jensen and *S. squarrosum* Crome. were selected for the study. At the end of July 2020, mature spore capsules of *Sphagnum angustifolium*, *S. magellanicum*, *S. squarrosum* and *S. fuscum* were collected from Tangbei, Hani and Dongfanghong peatlands, Northeastern China, respectively.

During collection, more than 20 shoots belonging to different populations in each peatland were chosen. Spore capsules were put in sealed PVC bottles in the field and later stored in a refrigerator at 4 °C in darkness before onset of the experiment.

Spore capsules of each species were surface sterilized with 75% ethanol for 2 min and were then crushed in a beaker with 6 mL distilled water to make a spore suspension. We put 2 mL of spore suspension into small bags made of polyamide filter fabrics with 7 µm mesh size.

### 4.2. Experimental Design

The whole experiment included two factors, heat and heat + smoke. There were three replicates and each with more than 300 spores for each species. In total, 96 spore bags (4 species × 8 treatments × 3 replicates) were prepared.

#### 4.2.1. Heat Treatments

In addition to the control treatment (20 °C: average daily temperature for the growing season in the Changbai Mountains), spore bags of each species were exposed to the following temperatures for 10 min: 40 °C, 60 °C and 100 °C [32]. The temperatures of 40 °C and 60 °C and exposure time of 10 min were chosen according to the soil temperature characteristics at different depths under the simulated burning conditions [32,59]. We chose 100 °C to represent a case of an extremely high temperature. The temperature treatments were performed in a pre-heated drying oven with stable heat settings and each replicate underwent the dry heat shock separately to guarantee its independence [38].

#### 4.2.2. Heat + Smoke Treatments

In this study, we performed an experiment combining heat followed by the exposure to smoke. In addition to the control, spore bags (each bag contains at least 300 spores) were exposed to 40 °C, 60 °C and 100 °C for 10 min.

*Sphagnum fuscum* shoots were collected to prepare a smoke-water solution by igniting its dry material (390 g) in a 3 L kettle [37]. The smoke was pumped into a PET bottle containing 300 mL of distilled water through a tube for two hours, and the prepared solution (pH 3.68 ± 0.01, *n* = 4) was considered as the 100% smoke–water (SW) stock solution [17,37]. In this experiment, we used the concentrations of smoke–water:distilled water (SW:DW, *v:v*) = 1:1 that stimulated best spore germination of peatland bryophytes [37]. Distilled water acted as the control. In addition to the control (imbibed in distilled water), heated spores were soaked in the smoke–water solution for 24 h and then put to germinate.

### 4.3. Germination Experiment

An improved Rudolph nutrient solution [30,60] was used as the culture solution. The culture solution and the agar powder were mixed and sterilized at a ratio of 50:1 to prepare the medium in Petri dishes. Spore suspension was poured evenly onto the medium for cultivation in a growth chamber (PRX-450C, Ningbo Saifu Experimental Instrument Co. Ltd., Ningbo, China). The cultivation conditions were 27 °C:22 °C under a 16 h:8 h, light:dark photoperiod, with light 60:0 μmol m^−2^ s^−1^, and a relative air humidity constant at 60% [31]. We observed spore germination twice, on days 11 and 21 of cultivation. The number of germinated (spores with filamentous protonema) and ungerminated spores were counted to produce a germination proportion.

### 4.4. Spore Viability Test

Viability of the spores was estimated by conducting methylene blue dyeing with three replicates of about 300 spores for each species [37,61]. Methylene blue is an alkaline dye which combines with nucleic acid to make the nucleus of viable spores blue [37]. Spores dyed blue were scored as alive while spores remaining undyed or only lightly dyed were scored as dead.

### 4.5. Data Processing and Statistical Analysis

#### 4.5.1. Data Processing

The spore germination percentage (GP), germination speed (GS), viability (V, dyeing percentage), and heat and heat + smoke treatment effect index (TEI) are calculated as follows:

GP (%) = (n/N) × 100%; GS = GP_11d_/GP_21d_; V (%) = (n_s_/N) × 100%.

where n is the number of germinated spores within a cultivation period; N is the total number of observed spores; n_s_ is the number of spores stained blue; GP_11d_ and GP_21d_ are GP at the 11th and 21st d, respectively.

When T ≥ C, TEI = 1 − C/T; When T < C, TEI = T/C − 1;

where C is the control group value and T is the treatment group value. When TEI > 0, the effect is promotive, and when TEI < 0, it is inhibitive. The absolute value of TEI represents the magnitude of the effect.

#### 4.5.2. Statistical Analysis

All statistical analyses were performed with SPSS for windows 26.0 (SPSS Inc., New York, NY, USA). We applied a two-way ANOVA to examine the main and interactive effects of species and heat on spore germination (GP) and spore viability (V). One-way ANOVAs were used to test the effect of heat singly and in combination with smoke on spore germination (GP), germination speed (GS) and treatment effect index (TEI) of each species. Tukey’s tests were performed to identify the difference in spore germination, viability, and treatment effect index among different species and treatments. The significance level was set to α = 0.05. Since the germination of all species under the treatment of 100 °C high temperature was zero, we did not use the data from this treatment when analyzing the effect of heat on spore germination.

## Figures and Tables

**Table 1:** Two-way analysis of variance (ANOVA) for the effect of species and heat temperature on germination percentage (GP) and spore viability (V, dyeing percentage) of *Sphagnum* species.

Index	Species		Heat		Species × Heat	
	*F*	*p*	*F*	*p*	*F*	*p*
GP	49.08	**<0.001**	23.58	**<0.001**	4.36	**0.004**
V	33.58	**<0.001**	165.39	**<0.001**	14.52	**<0.001**

Bold values indicate statistically significant effects (*p* < 0.05).

**Table 2:** One-way analysis of variance (ANOVA) for the effect of heat singly and combined with smoke on spore germination percentage (GP), germination speed (GS) and treatment effect index (TEI) of four *Sphagnum* species.

Species	Index	Heat		Heat + Smoke	
		*F*	*p*	*F*	*p*
*S. angustifolium*	GP	8.67	**0.017**	27.75	**<0.001**
	GS	1.55	0.287	4.23	**0.046**
	TEI	6.90	**0.028**	10.48	**0.004**
*S. fuscum*	GP	5.66	**0.042**	8.58	**0.007**
	GS	6.50	**0.031**	2.34	0.150
	TEI	1.702	0.260	3.03	0.093
*S. magellanicum*	GP	3.47	0.100	23.14	**<0.001**
	GS	0.10	0.904	2.70	0.116
	TEI	4.85	0.056	14.12	**0.001**
*S. squarrosum*	GP	19.70	**0.002**	20.42	**<0.001**
	GS	0.738	0.517	1.73	0.238
	TEI	34.88	**<0.001**	36.25	**<0.001**

Bold values indicate statistically significant effects (*p* < 0.05).

## Data Availability

The data presented in this study are available in the article. Additional data are available on request from the corresponding author.
